# An RBD bispecific antibody effectively neutralizes a SARS-CoV-2 Omicron variant

**DOI:** 10.1186/s44280-023-00012-0

**Published:** 2023-04-30

**Authors:** Mengqi Yuan, Yanzhi Zhu, Guanlan Liu, Yujie Wang, Guanxi Wang, Guozhong Zhang, Lilin Ye, Zhaohui Qian, Pinghuang Liu

**Affiliations:** 1 Key Laboratory of Animal Epidemiology of the Ministry of Agriculture, College of Veterinary Medicine, China Agricultural University, Beijing, 100193 China; 2 College of Biological Sciences, China Agricultural University, Beijing, 100193 China; 3 Institute of Immunology, PLA, Third Military Medical University, Chongqing, 400038 China; 4 NHC Key Laboratory of Systems Biology of Pathogens, Institute of Pathogen Biology, Chinese Academy of Medical Sciences and Peking Union Medical College, Beijing, 100176 China

**Keywords:** SARS-CoV-2, Bispecific antibodies, Neutralizing antibodies, Escape variants, Omicron variants

## Abstract

Potent neutralizing antibodies (nAbs) against SARS-CoV-2 are a promising therapeutic against the ongoing COVID-19 pandemic. However, the continuous emergence of neutralizing antibody escape variants makes it challenging for antibody therapeutics based on monospecific nAbs. Here, we generated an IgG-like bispecific antibody (bsAb), Bi-Nab, based on a pair of human neutralizing antibodies targeting multiple and invariant sites of the spike receptor binding domain (RBD): 35B5 and 32C7. We demonstrated that Bi-Nab exhibited higher binding affinity to the Delta spike protein than its parental antibodies and presented an extended inhibition breadth of preventing RBD binding to angiotensin-converting enzyme 2 (ACE2), the cellular receptor of SARS-CoV-2. In addition, pseudovirus neutralization results showed that Bi-Nab improved the neutralization potency and breadth with a lower half maximum inhibitory concentration (IC_50_) against wild-type SARS-CoV-2, variants being monitored (VBMs) and variants of concern (VOCs). Notably, the IgG-like Bi-Nab enhanced the neutralizing activity against Omicron variants with potent capabilities for transmission and immune evasion in comparison with its parental monoclonal antibody (mAb) 32C7 and a cocktail (with the lowest IC_50_ values of 31.6 ng/mL against the Omicron BA.1 and 399.2 ng/mL against the Omicron BA.2), showing evidence of synergistic neutralization potency of Bi-Nab against the Omicron variants. Thus, Bi-Nab represents a feasible and effective strategy against SARS-CoV-2 variants of concern.

## Introduction

The emerging COVID-19 global outbreak has had widespread effects, and its causative agent, SARS-CoV-2, continues to evolve. Neutralizing mAbs that target the RBD of the SARS-CoV-2 spike protein are reported as the most promising antibody-based therapeutics against SARS-CoV-2 infection [1–3]. However, the numerous emerging genetically distinct SARS-CoV-2 VOCs have become the major concern of the COVID-19 pandemic, particularly the more transmissible and more immune evasive VOCs with substantial mutations, such as the Delta variant and the Omicron variant, which undermine the efficacy of existing vaccines and therapeutic neutralizing antibodies. The Delta and Omicron variants have rapidly become global dominant variants since their emergence [4–6]. As SARS-CoV-2 continues to mutate and evolve, the World Health Organization (WHO) has announced twelve variants since the beginning of the COVID-19 pandemic, which are classified as VBMs (including Alpha, Beta, Gamma, Epsilon, Eta, lota, Kappa, 1.617.3, Mu and Zeta) and VOCs (including Delta and Omicron) [7–9]. Alpha (also known as B.1.1.7) and Beta (also known as B.1.351), as earlier variants, are refractory to neutralization by many mAbs targeting the RBD and N-terminal domain (NTD) [10–12]. Alpha harbors an N501Y mutation in the RBD, leading to enhanced transmissibility [13]. Eta carries additional K417N and E484K mutations in the RBD along with the N501Y and increases binding affinity to ACE2 [14, 15]. Among these variants, Omicron rapidly became dominant worldwide since it was first described in November 2021 [16, 17]. The Omicron variant is characterized by more than thirty spike amino acid mutations, and most of them are located in the RBD, leading to the decreased efficacy of currently available vaccines and therapeutic mAbs for clinical use, including casirivimab, bamlanivimab and regdanvimab [12, 15, 18–20]. The previously dominant Delta variant (B.1.617.2) that harbors L452R and E484Q mutations is more resistant to neutralization by COVID-19 convalescent patient sera [7]. These results highlight the importance of developing a more effective strategy that potently neutralizes VBMs and VOCs.

With the emergence of new SARS-CoV-2 variants that are resistant to many existing neutralizing mAbs, it is favorable to develop multiple specific antibodies or the combination of multiple neutralizing antibodies. To date, multiple studies have reported that cocktails of two or three mAbs can achieve satisfactory results in a synergistic way compared to a single antibody, limiting the possibility of virus escape [21, 22]. However, what follows is the increased manufacturing costs and volume. The bsAb strategy takes advantage of two distinct mAbs that target two antigen-binding sites with one molecule and is widely used in cancer and inflammatory treatment [23, 24].

RBD-targeting neutralizing mAbs 35B5 and 32C7 were isolated from COVID-19 convalescent patients. In vitro and in vivo, human mAb 35B5 can efficiently neutralize WT SARS-CoV-2, Delta and Omicron by targeting a unique conserved epitope that is distinct from those of the four identified classes of neutralizing antibodies to RBD [25]. In this study, we generated an IgG-like bsAb Bi-Nab based on 35B5 and 32C7 targeting the RBD domain of the spike protein, potently neutralizing the emerging SARS-CoV-2 Omicron variant. Our results show that Bi-Nab increased the binding affinity with the spike protein among WT SARS-CoV-2 and SARS-CoV-2 variants. Notably, Bi-Nab has an improved neutralization activity against the immune evasion Omicron variant compared to parental mAbs and a cocktail. Thus, as a new antibody-based tool, bsAb is an effective strategy in protecting against mutational virus escape.

## Results

### Design and expression of IgG-like Bi-Nab

**Fig. 1:**
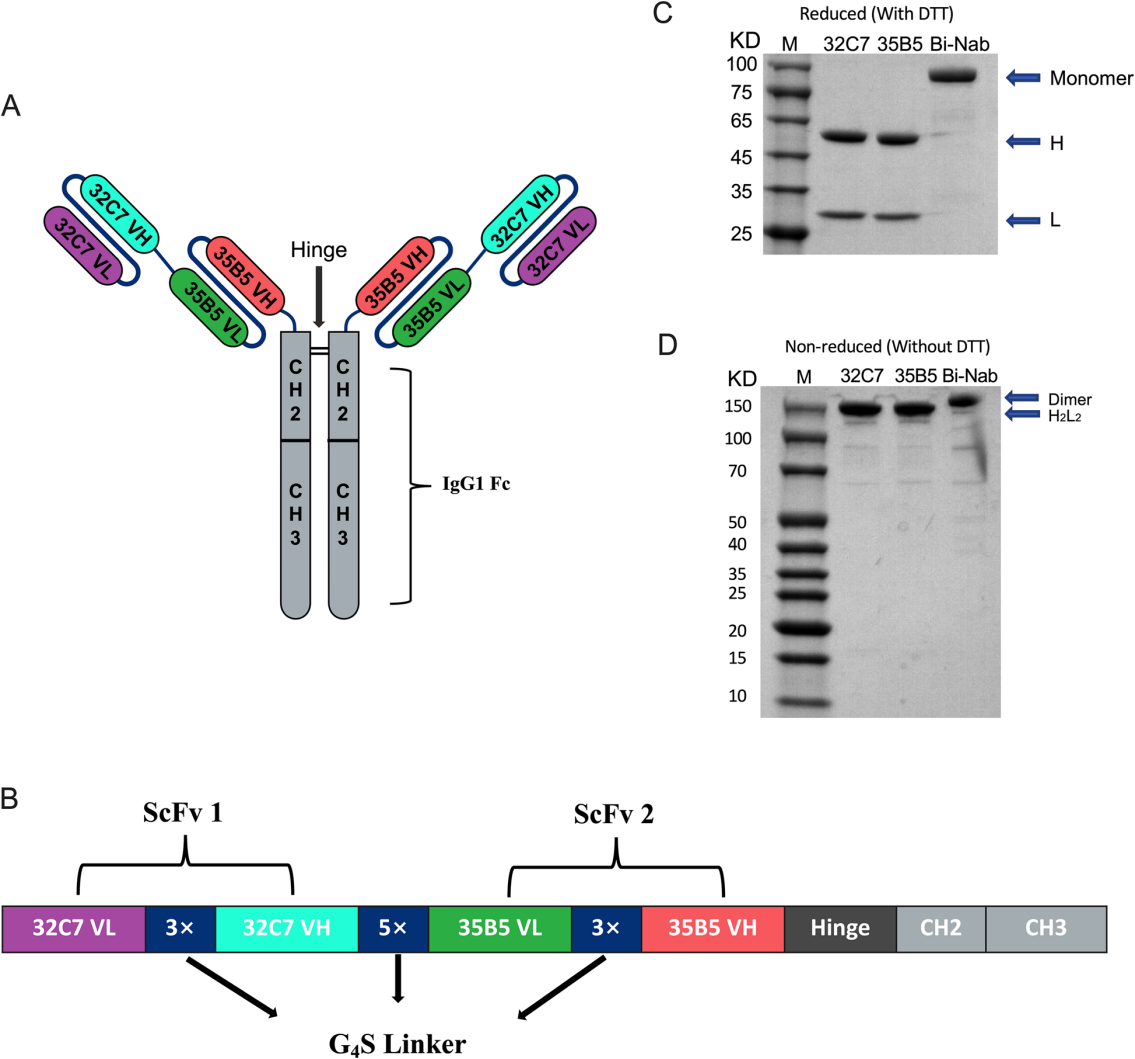
Construction and generation of the bispecific antibody. **A** Schematic diagram of the molecular configurations of IgG-like bsAb Bi-Nab. **B** Schematic presentation of the IgG-like bispecific antibody Bi-Nab. The variable regions of the heavy and light chains of 32C7 or 35B5 are color-coded (the variable light chain and heavy chain of 32C7 are colored purple and light blue, respectively; the variable light chain and heavy chain of 35B5 are colored green and light red, respectively); human IgG1 Fc is colored gray. The 3 × G4S and 5 × G4S linkers were used to form the scFv 32C7-35B5. **C** The equal mass of Bi-Nab or two parental mAbs was analyzed by SDS‒PAGE under reducing conditions with DTT. H and L denote the heavy and light chains of parental mAbs, respectively. The molecular weight of the Bi-Nab monomer is 90 kD. Antibodies were expressed in ExpiCHO-S cells and captured on Protein-A affinity resin. M, molecular weight standard. **D** Nonreduced SDS‒PAGE analysis of Bi-Nab or two parental mAbs. The antibodies were analyzed under direct affinity elution conditions without DTT, and three independent experiments were performed with the same results

We sought to construct an IgG-like bsAb that targets RBD to combine the utility of two identified mAbs, 35B5 and 32C7. The mAb 35B5, a new class RBD-targeting antibody isolated from convalescent COVID-19 patient memory B cells, showed potent neutralizing activities against Omicron and other VOCs by targeting a unique RBD epitope [26]. The mAb 32C7, a classic class 3 family of neutralizing antibodies, neutralizes multiple SARS-CoV-2 variants [27]. Given that these two neutralizing mAbs bound to distinct RBD domains, we assessed possible synergy when combining them. We used the IgG-(ScFv)_2_ design and engineered an IgG-like bsAb Bi-Nab in the tetravalent format (Fig. 1A). A 3X G_4_S linker was used to connect the heavy chain and light chain, with the first antibody 32C7 making up the ScFv1 domain and the second antibody 35B5 making up the ScFv2 domain close to the Fc. To prevent possible steric resistance, a 5X G_4_S linker was made to connect the individual ScFvs to form the 32C7_VL-VH_ → G_4_S linker → 35B5_VL-VH_ domain fused to the human IgG1 Fc (Fig. 1B). The IgG-like form of a bsAb has a similar structure to native IgG and is easy to express. The bsAb Bi-Nab was produced by transient expression in ExpiCHO-S cells followed by Protein A chromatography purification. The purified Bi-Nab, with the expected molecular weight (monomer weight at 90 kD, dimer weight at 180 kD), was correctly assembled as analyzed by reducing and nonreducing SDS‒PAGE (Fig. 1C and D).

### Enhanced antigen binding and RBD-ACE2 interaction blocking by Bi-Nab

**Fig. 2:**
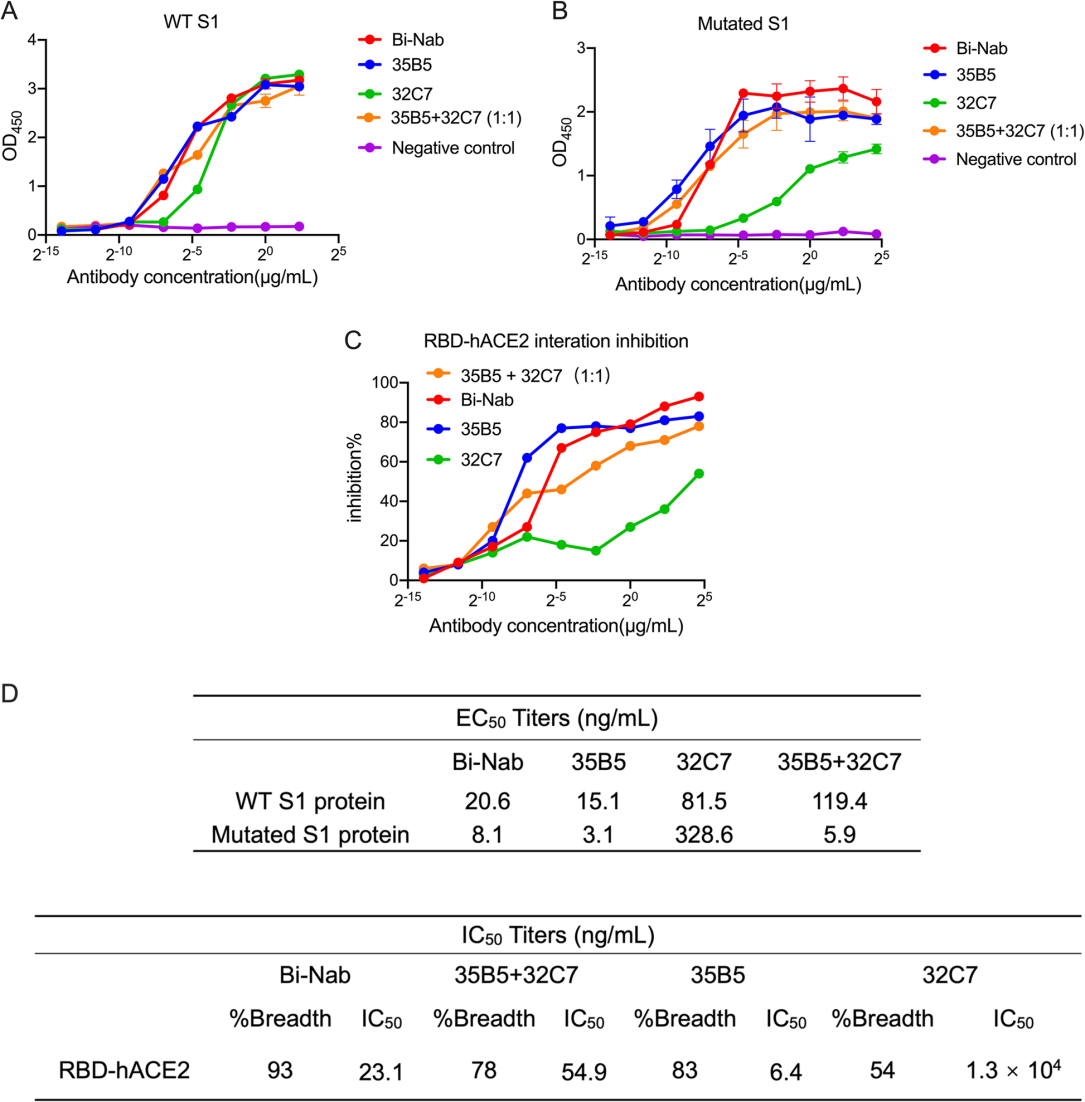
Determination of EC_50_ and IC_50_ by ELISA. **A** ELISA-based WT SARS-CoV-2 S1 (fusion protein with His tag) binding assay of Bi-Nab. **B** ELISA binding assay of Bi-Nab to mutated SARS-CoV-2 S1 (fusion protein with a His tag). The EC_50_, the concentration for 50% of the maximal effect, is used as a parameter for assessing the affinity between antibodies and spike proteins. **C** ELISA analysis of Bi-Nab or parental mAbs or cocktail-mediated inhibition of WT RBD protein (fusion protein with mouse Fc part) binding to human ACE2. The IC_50_, the half-maximal inhibitory concentration, was determined by sigmoidal dose‒response nonlinear regression. **D** The ELISA statistics of Bi-Nab, a cocktail, and parental mAbs are shown in the tables

Next, we performed an ELISA binding assay to determine the affinity of the RBD-targeting Bi-Nab to the SARS-CoV-2 RBD. We found that Bi-Nab retained binding affinity to both WT S1 proteins and mutated S1 proteins with concentrations for EC_50_ values of 20.6 ng/mL (Fig. 2A) and 8.1 ng/mL (Fig. 2B), respectively. Bi-Nab had a high affinity (< 100 ng/mL) to the WT RBD (Fig. 2A), similar to its parental mAb 35B5, notably, better than the mAb cocktail (Fig. 2A). 32C7, with a good affinity for the WT RBD in the panel, showed a significant decrease in affinity for mutated S1 of approximately fourfold with an EC_50_ value of 328.6 ng/mL (Fig. 2D). These results suggest that the N501Y mutation in the RBD may play an important role in affecting the binding of 32C7 to RBD but has no effect on Bi-Nab. In parallel, to evaluate whether Bi-Nab maintains blocking activity against RBD binding to ACE2 just as their parental mAbs do, we performed an ELISA-based receptor-binding inhibition of hACE2 assay. Bi-Nab blocked the RBD and ACE2 interaction with an IC_50_ value of 23.1 ng/mL, which is slightly higher than the IC_50_ of 35B5 (6.4 ng/mL) and lower than the IC_50_ of the mAb cocktail (54.9 ng/mL) (Fig. 2C). Notably, the inhibition potency of Bi-Nab was extended to 93%, indicating the improvement of inhibiting activity over parental mAbs. These results suggest that the construction of bsAb Bi-Nab contributes to the good binding affinity to RBD and the improvement of blocking activity for Bi-Nab over parental mAbs.

### Bi-Nab binds the spike protein of most SARS-CoV-2 variants

**Fig. 3:**
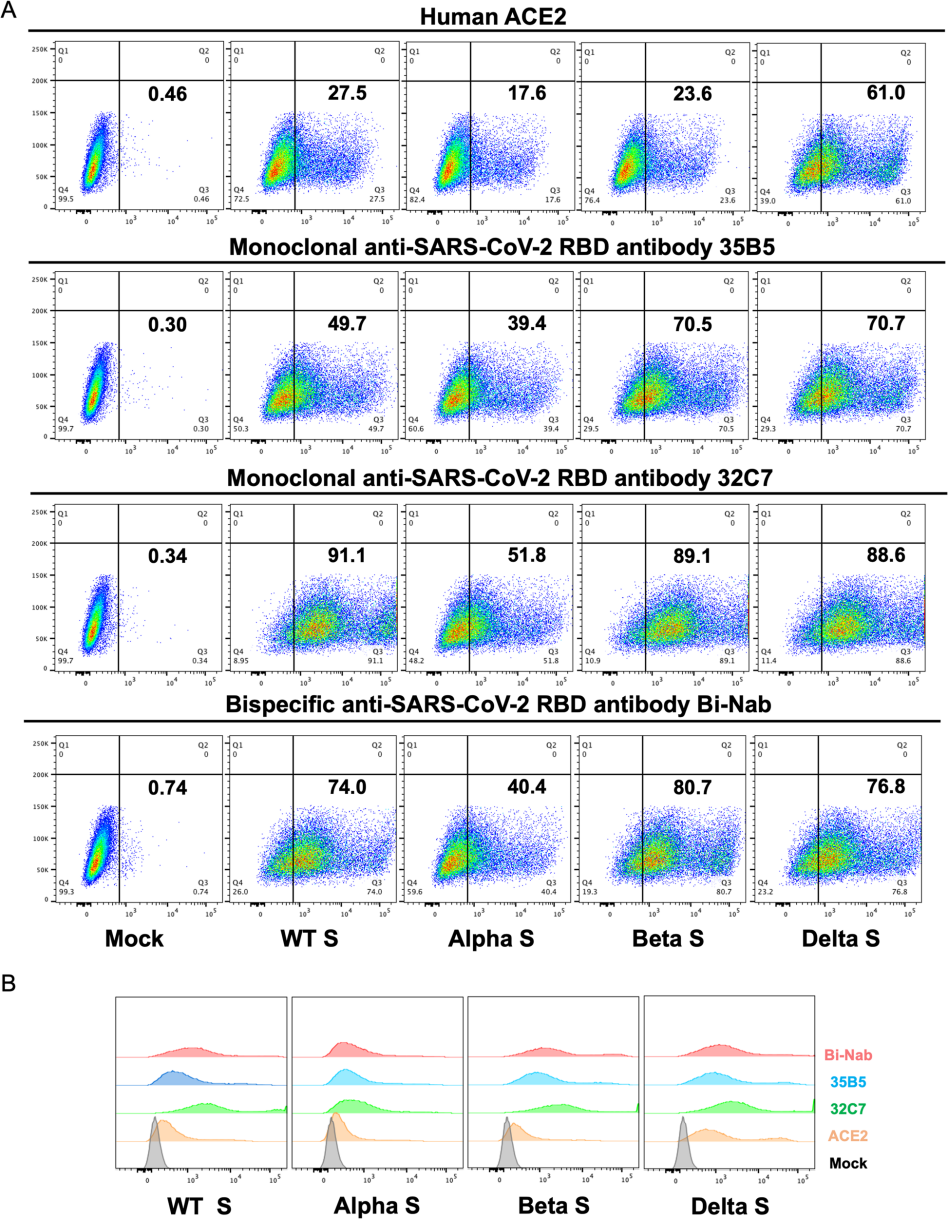
Assessment of the binding affinity of Bi-Nab to the SARS-CoV-2 S protein. **A** HEK293T cells separately transfected with plasmids encoding WT SARS-CoV-2 S, Alpha S, Beta S, or Delta S proteins were incubated with Bi-Nab, 32C7 or 35B5, respectively, followed by a FITC-conjugated anti-human IgG antibody or FITC-conjugated anti-His antibody. As a positive control, hACE2 with a His-tag was used. The binding affinity was quantified using flow cytometry. Mean fluorescence intensity (MFI) was normalized to the mock (empty vector) group. **B** The fluorescence mobility of Bi-Nab binding to Delta S was significantly improved. The experiments were performed three times, and one representative is shown

Next, we performed the binding of Bi-Nab to the cellular surface-expressing spike protein of SARS-CoV-2 WT, Alpha variant (with a single mutation N501Y in RBD), Beta variant (with K417N, E484K, and N501Y mutations in RBD), and Delta variant (with L452R and T478K mutations in RBD) in native conformation by flow cytometry assay to evaluate the binding affinity of these three antibodies to spike protein. The results revealed that Bi-Nab showed a strong affinity for SARS-CoV-2 variant spike proteins, similar to their parental mAbs (Fig. 3A). Among the four SARS-CoV-2 strains, Bi-Nab, and parental mAbs easily detected the expressed spike protein of WT, Beta, and Delta on the HEK293T cell surface but with a significant decrease in the Alpha spike protein (Fig. 3B). Consistent with the ELISA binding results, the affinity of 32C7 for mutant S1 was significantly reduced, indicating that the N501Y mutation may play a critical role in the affinity of RBD-targeting antibodies to RBD by inducing conformational changes in the spike protein. Notably, the binding capacity of both ACE2 and Bi-Nab to the Delta variant spike protein was increased (Fig. 3B). Consistent with a previous report, the two substitutions L452R and T478K in the Delta RBD participate in the hACE2 interaction and enhance the affinity of the interaction between them (Fig. 3) [25]. Taken together, Bi-Nab, combining two distinct RBD targeting mAbs 35B5 and 32C7, shows good affinity to the native spike proteins of SARS-CoV-2 WT, Beta, and Delta and slightly to Alpha, although not significantly higher than either parental mAb 32C7 alone.

### Broader neutralization of Bi-Nab to SARS-CoV-2 VBMs

**Fig. 4:**
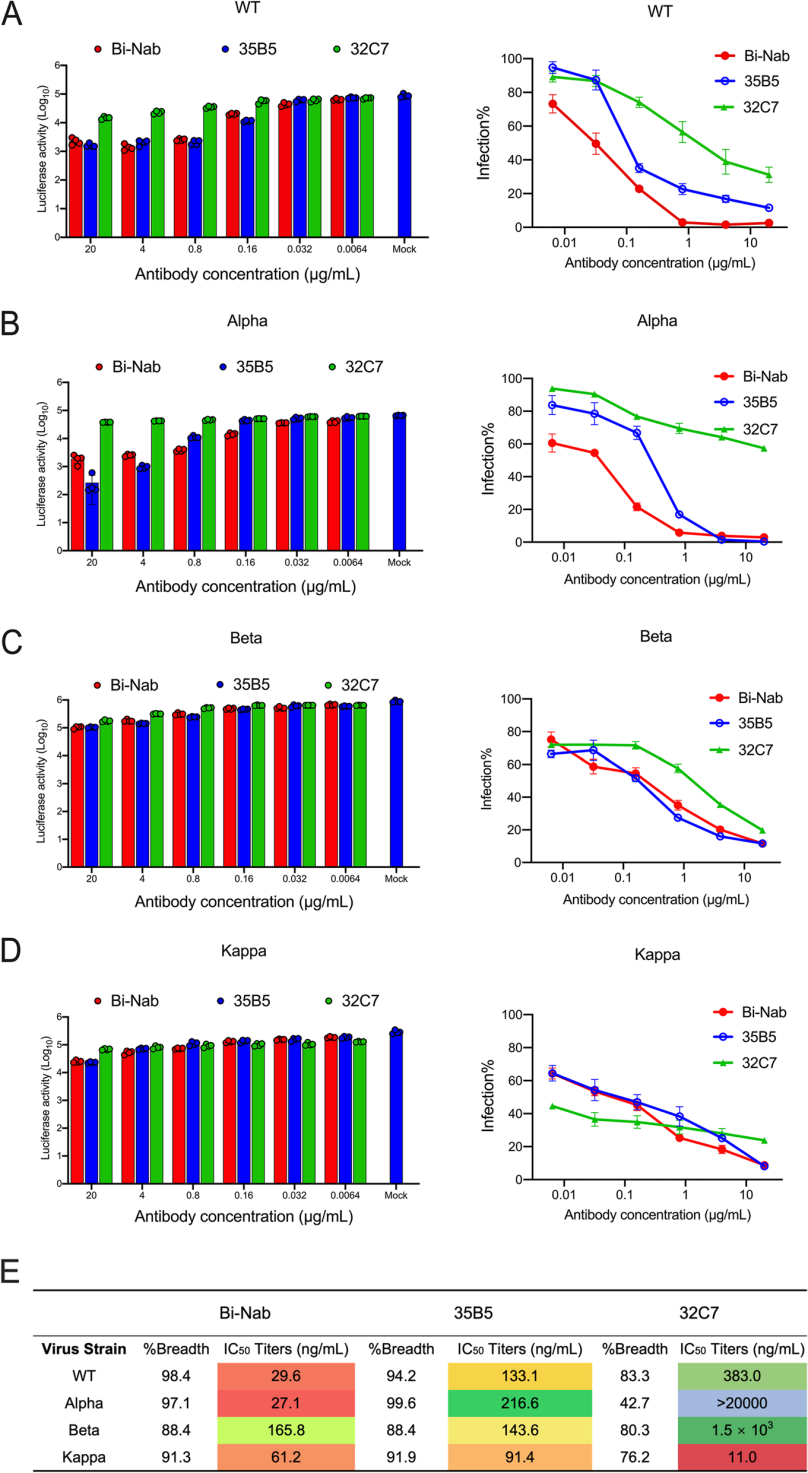
Broad neutralization of Bi-Nab against WT and VBM pseudoviruses. The neutralization breadth of Bi-Nab or parental antibodies was tested against a SARS-CoV-2 pseudovirus panel consisting of spikes of WT (**A**), Alpha (**B**), Beta (**C**), and Kappa (**D**) VBM strains. Heatmaps of IC_50_, breadth and potency are shown in (**E**), and warmer colors were used to indicate more potent neutralization. The neutralization breadth of an antibody against SARS-CoV-2 VBMs was based on the maximum percentage of pseudovirus that can be neutralized by that antibody. The IC_50_ values were determined by a nonlinear four-parameter dose‒response curve. Error bars represent the mean values ± SEMs

Emerging SARS-CoV-2 VBMs, such as B.1.1.7 (Alpha), B.1.351 (Beta), and B.1.617 (Kappa, with L452R and E484Q mutations in RBD), harbor mutations that may decrease the efficacy of therapeutic mAbs. To determine the neutralization potency of Bi-Nab, we performed a lentiviral-based SARS-CoV-2 pseudovirus neutralization assay against WT SARS-CoV-2 and VBMs, including Alpha, Beta, and Kappa variants. Bi-Nab significantly neutralized WT and Alpha pseudoviruses with the lowest IC_50_ values of 29.6 ng/mL (Fig. 4A) and 27.1 ng/mL (Fig. 4B), respectively. Meanwhile, Bi-Nab showed enhanced neutralization potency against Beta in comparison with mAb 32C7 with an IC_50_ value of 165.8 ng/mL (Fig. 4C), consistent with the previous finding that the Beta variant exhibits considerable escape of the most potent neutralization monoclonal antibodies. The decreased neutralizing activity of Bi-Nab and parental mAb 32C7 against Beta pseudovirus was observed and summarized as fold decreases in IC_50_ relative to the WT pseudovirus (Fig. 4C and E) [11, 12, 28]. Bi-Nab neutralized Kappa pseudovirus with an IC_50_ value of 61.2 ng/mL, which is lower than the IC_50_ value of 35B5 (91.4 ng/mL) and is higher than that of 32C7 (11.0 ng/mL) (Fig. 4D and E). The three antibodies showed high affinity for the Beta spike and low affinity for the Alpha spike by flow cytometry analysis, but the pseudovirus neutralization results were the exact opposite, suggesting that high affinity for the spike in the native conformation may be necessary but not sufficient for neutralization in the context of the virus. Collectively, the pseudovirus neutralization results of bsAb Bi-Nab showed enhanced cross-reactivity among WT SARS-CoV-2, Alpha, Beta, and Kappa variants in comparison with their parental mAbs 35B5 and 32C7 and highlight the importance of combination antibody therapy to address the expanding SARS-CoV-2 variants.

### Bi-Nab improves neutralization against Omicron variants

**Fig. 5:**
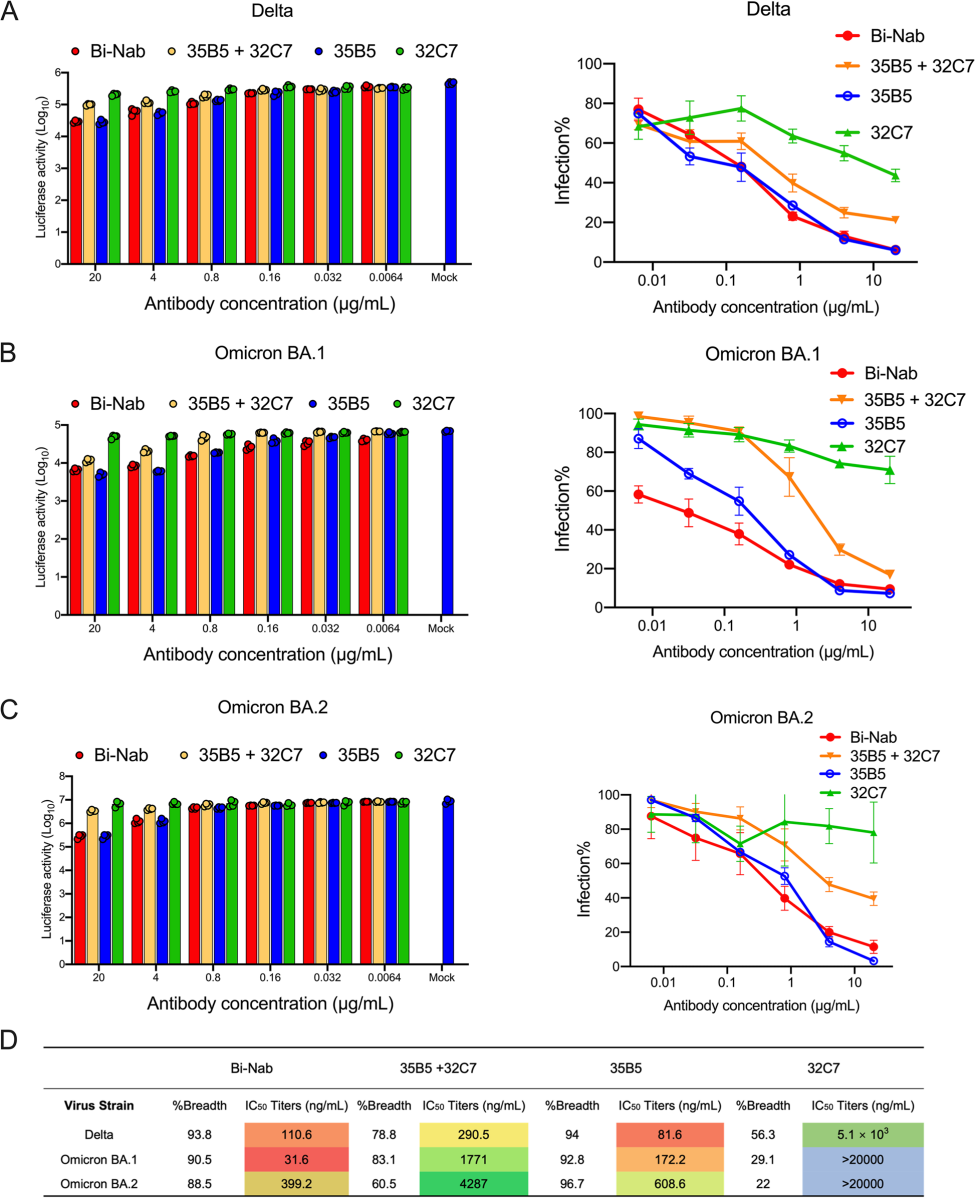
Bi-Nab demonstrates potent neutralizing activity against Omicron VOCs. The neutralization breadth and potency of Bi-Nab, a cocktail, and parental antibodies against Delta (**A**), Omicron BA.1 (**B**), and Omicron BA.2 (**C**) pseudoviruses were evaluated using a lentiviral-based pseudovirus system. **D** Heatmaps of IC_50_, breadth and potency are shown in Excel, and warmer colors were used to indicate more potent neutralization. The neutralization breadth of an antibody against SARS-CoV-2 VOCs was based on the maximum percentage of pseudovirus that can be neutralized by that antibody

Compared to the mutations found in other variants, the Omicron lineage has more than thirty amino acid mutations in the spike protein, with the majority of alterations structurally focused in the receptor-binding motif (RBM) of RBD, in locations accessible to antibodies near the top of the spike, increasing the risk of immune evasion [29, 30]. In line with previous reports [27], the pseudovirus neutralization assay indicated that 32C7, which targets the RBD but does not block ACE2 binding, could not efficiently neutralize SARS-CoV-2 VOCs and only exhibited substantially lower but detectable neutralization against Delta (with an IC_50_ of 5.1 μg/mL) (Fig. 5A), as 32C7 is a classic class 3 neutralizing antibody that neutralizes SARS-CoV-2 via the Fc-mediated effector mechanism [27]. In comparison with 35B5 and the cocktail, Bi-Nab showed enhanced neutralization potency against Omicron BA.1 and Omicron BA.2 with the lowest IC_50_ values of 31.6 ng/mL and 399.2 ng/mL, respectively (Fig. 5B and C). Surprisingly, compared with the less potent parental mAb 32C7, Bi-Nab still had a relatively high affinity against Delta and Omicron variants, with an IC_50_ range of 100–400 ng/mL (Fig. 5D), suggesting the elevated potency of bsAb Bi-Nab in protecting against the mutational virus escape, especially for the Omicron variants.

### Structural basis for tight binding and potent neutralization against VBMs and VOCs

**Fig. 6:**
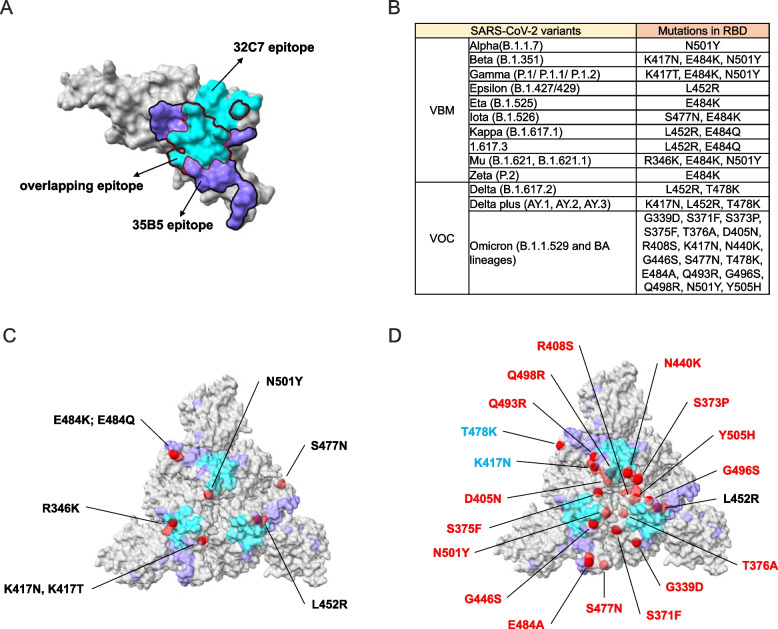
Structural explanations of the effect of critical mutations on Bi-Nab activity. **A** The epitopes for 32C7 and 35B5 on the surface of RBD are mapped. The epitopes for 32C7 and 35B5 (with black contours) on the RBD are colored blue and purple, respectively. The overlapping epitopes are mapped with red contours. **B** Mutations that have been reported in the RBD region of VBMs and VOCs are shown in Excel. **C** Mutations in the RBD of VBMs in (**B**) are indicated by red spheres on the surface representation of the S trimer. **D** Mutations in the RBD of VOCs indicated in (**B**) as red spheres on the S trimer structure, on which the epitopes of 32C7 (blue) and 35B5 (purple) are shown. The structure was visualized by PyMol (Schrodinger: https://www.schrodinger.com/pymol)

To investigate the structural basis of Bi-Nab potently neutralizing SARS-CoV-2 VBMs and VOCs, a structural explanation of how Bi-Nab exhibits tight binding and potent neutralization against VBMs and VOCs is presented in Fig. 6. Both 32C7 and 35B5 are RBD-targeting neutralizing antibodies with epitopes on RBD opposite to the ACE2-binding surface, but 32C7 covers a much smaller epitope surface on RBD than that of 35B5, with thirteen overlapping residues between the epitopes of two mAbs in RBD including T345, R346, F347, A348, S349, Y351, A352, N354, K444, N450, Y451, L452, and R466 (Fig. 6A). Compared to the mutations found in VBMs, VOCs harbor more mutations that are structurally focused in RBD [25]. These mutations were summarized and classified according to the SARS-CoV-2 genetic lineages (Fig. 6B). The SARS-CoV-2 VBMs involved in this work contain several prevailing mutations in the RBD, including N501Y (Alpha and Beta), K417N (Beta), L452R (Kappa) and E484K/E484Q (Beta and Kappa). Structural analysis shows that residues N501 and K417 are not involved in Bi-Nab-RBD interactions. Consistently, Bi-Nab has neutralization efficacy comparable to that of the Alpha pseudovirus and WT (Fig. 4A and B). Specifically, residues L452R and E484K/E484Q are located on the epitope of the Bi-Nab-RBD interface, whereas E484K/E484Q are at the edge of the 35B5 epitope, and L452R is confirmed to not affect the binding affinity of 35B5 to RBD (Fig. 6C) [25]. Consistent with the slightly decreased neutralization efficacy to the Kappa variant and obviously decreased neutralization efficacy to the Beta variant, indicating that E484K might disrupt the binding of Bi-Nab to RBD (Fig. 4C and D).

The Omicron VOCs are characterized by more than thirty mutation sites in the spike, with most focus on the RBD-induced significant structural changes (Fig. 6B). Among these mutation sites in the Omicron RBD, G339D, G446S, and E484A are located at the edge of the Bi-Nab-RBD interface, and the others are located far from the epitope for Bi-Nab (Fig. 6D). Meanwhile, only three mutation sites, K417N, L452R and T478K, are characterized in the Delta RBD. Specifically, K417 and N460 are not located on the epitope of Bi-Nab, whereas L452 is at the edge of the Bi-Nab epitope and confirmed that it does not affect the binding affinity of 35B5 to the RBD domain. Thus, structural analysis supported the neutralizing and binding results and suggested that the fine specificity of the antibody epitopes is critical for the effective neutralization of SARS-CoV-2.

## Discussion

The continuously emerging new SARS-CoV-2 variants, particularly the more transmissible and more immune-evasive VOC Delta and Omicron variants, have undermined the efficacy and effectiveness of existing vaccines and therapeutic neutralizing antibodies [29, 31, 32]. This highlights the need to develop additional antibody strategies against viral evasion. MAb cocktails or multispecific antibodies are a favorable strategy to combat highly mutated SARS-CoV-2 variants [21, 33, 34]. Nevertheless, we did not observe unequivocal synergy among the SARS-CoV-2 specific mAb cocktail, consistent with the lack of research demonstrating true synergy among these mAbs [3]. We explored to using the IgG-like bispecific antibodies as a potential succedaneum to antibody cocktails. The bsAb is more cost-effective and convenient than antibody cocktails, indicating there is a high potential to develop the bsAb as an alternative to antibody cocktails. IgG-like bsAbs have been widely used in the development of clinical drugs due to their stable structure and long half-life [35]. Here, we designed a bispecific antibody Bi-Nab that potently neutralizes the SARS-CoV-2 Omicron variant by targeting multiple and invariant sites of the RBD. Bi-Nab demonstrated retained the capacity to neutralize WT, Alpha, Beta, Kappa, Delta, and Omicron pseudoviruses at doses tested in vitro, so future experiments should be performed to evaluate Bi-Nab against authentic SARS-CoV-2 in vitro and in vivo.

The Omicron variant is more immune evasive and contagious than earlier variants of SARS-CoV-2, including the Delta variant, and has become the dominant strain across the world [36, 37]. The Omicron variant is characterized by extensive mutations in the spike and harbors 15 mutations in the RBD, which includes N501Y, G496S, K417N, Q493R, and G446S in the loop regions of the ACE2 binding motif [26, 29]. This is why Omicron escapes most of the current RBD neutralizing antibodies. The Omicron variant also harbors G339D and N440K mutations located in the epitope of class 3 SARS-CoV-2 antibodies [1, 38–40]. Thus, the neutralization of the class 3 neutralization mAb 32C7 against Omicron was severely blunted (Fig. 5D). In addition, the mutations of D796Y, L981F, and N969K in Omicron S2 enhance intratrimer interactions and trimer stability by increasing hydrophobic and hydrogen-bond interactions in S2 units [26].

Unlike most SARS-CoV-2 class 3 neutralizing antibodies, 35B5 contains a few residues of the epitopes of them. These overlapping invariant epitopic residues are far from the mutation sites in the Omicron RBD. In addition, 35B5 neutralizes SARS-CoV-2 by disrupting the spike trimer structure rather than directly interfering with ACE2 binding [26]. Compared with WT, G339D, G446S, and E484A among the 15 Omicron RBD mutations reduced the sensitivity of the Omicron variant to 35B5 since they are located at the edge of 35B5 and the RBD interface though no structural collision with 35B5 Fab. As expected, the cocktail of 32C7 and 35B5 did not synergize their neutralization activity against Omicron instead of interferent activity since they both target RBD and have overlapping binding areas in the RBD (Fig. 5). Notably, Bi-Nab combined with 35B5 and 32C7 displays enhanced neutralization activity relative to their parental mAb cocktails or alone. Interestingly, although the Omicron, Alpha, Beta, and Delta variants completely escape 32C7 neutralization (Figs. 4B-C and 5), 32C7 shows the best binding affinity among Alpha, Beta, and Delta variants (Fig. 3), suggesting that the binding activity of antibodies to cellular surficial spike is not correlated with neutralization activity. The higher spike binding activity of 32C7 relative to 35B5 makes 32C7 outcompete in binding to SARS-CoV-2 pseudoviruses in the cocktail, which may be the reason why the cocktail has less neutralization against Delta and Omicron variants compared with Bi-Nab (Fig. 5).

## Conclusions

In summary, we designed an RBD bispecific antibody that potently neutralizes a range of SARS-CoV-2 variants, including the currently dominant Omicron variant. Bi-Nab displayed a greater neutralizing potency than its parental antibodies against SARS-CoV-2 VBMs and VOCs. Our data indicate that bispecific antibodies provide a feasible strategy against emerging SARS-CoV-2 variants in the future.

## Materials and methods

### Cells and plasmids

HEK293 and HEK293T cells were cultured in Dulbecco’s modified Eagle’s medium (DMEM) (Gibco, USA) supplemented with 10% fetal bovine serum (FBS) (Gibco, USA) and 1% penicillin‒streptomycin and maintained at 37 °C in a 5% CO_2_ setting. HEK293 and HEK293T cells transiently expressed recombinant human ACE2 (293/hACE2) or produced pseudoviruses, respectively. ExpiCHO-S cells, adapted to high-density suspension culture in ExpiCHO™ expression medium, were passaged every 3–4 days at a ratio of 1:20 when cultures reached a density of 4 × 10^6^ viable cells/mL in shaker flasks. The flasks were incubated on a 37 °C or bital shaker platform with ≥ 80% relative humidity and 8% CO_2_. The plasmid encoding Bi-Nab was constructed by molecular cloning methods and verified by sequencing. The pcDNA3.1-hACE2 plasmid with human codon optimization, as well as plasmids encoding WT SARS-CoV-2 spike protein, SARS-CoV-2 VBM spike protein, SARS-CoV-2 VOC spike protein, lentiviral packaging plasmid psPAX2 and pLenti-GFP lentiviral reporter plasmid, were kindly provided by Dr. Zhaohui Qian from the Chinese Academy of Medical Sciences and Peking Union Medical College.

### Design, expression, and purification of bispecific antibody

SARS-CoV-2 RBD specific mAbs 35B5 and 32C7, isolated from WT SARS-CoV-2 infected patients’ memory B cells, were used to create the IgG-like bsAb Bi-Nab. The DNA sequence was obtained by synthesizing plasmids that had the heavy chains or the light chains of two antibodies in tandem, as previously described [41, 42]. First, we connected the sequences of the variable domains of mAbs 35B5 and 32C7 via tandem glycine-glycine-glycine-glycine-serine (G4S) peptide linkers to generate a single-chain Fv (ScFv) format 32C7-35B5. Specifically, the variable regions of heavy and light chains of 35B5 or 32C7 were connected by a 3 × G4S linker, and then a 5 × G4S linker was used to connect the sequences of the variable domains of 35B5 and 32C7. The codon-optimized ScFv DNA sequences were synthesized by GenScript (Piscataway, NJ, China) followed by cloning into the eukaryotic cell expression vector AbVec-hIgG1 backbone digested with AgeI (New England Biolabs, USA) and Hind III (New England Biolabs) to construct ORF of IgG-like bsAb Bi-Nab. Bi-Nab was produced by using the ExpiCHO™ Expression System (A29133, Gibco, USA) following the manufacturer’s instructions and stored at −80 °C after purification by Protein-A affinity resin (L00210-10, GenScript, NJ, China).

### Antibodies and antigens

Horseradish peroxidase (HRP)-conjugated anti-mouse IgG antibody (ab97265, UK) and HRP-conjugated anti-human IgG antibody (ab99759) were purchased from Abcam. His-tagged rabbit mAb (Alexa Fluor® 488 Conjugate) was purchased from Cell Signaling Technology (#14,930, USA), and FITC-conjugated anti-human IgG was purchased from ZSGB-Bio (ZF-0308, BJ, China). SARS-CoV-2 S1 protein of WT strain (40,591-V08H, BJ, China), his recombinant protein SARS-CoV-2 S1 with HV69-70 deletion, N501Y and D614G (40,591-V08H7), SARS-CoV-2 S2 (40,590-V08B) protein, SARS-CoV-2 RBD protein (40,592-V08B) and his-tag hACE2 protein (10,108-H08H) were purchased from Sino Biological.

### Enzyme-linked immunosorbent assay (ELISA)

The binding ability of antibodies to WT SARS-CoV-2 S1 protein or mutated SARS-CoV-2 S1 protein was assessed by an ELISA with modifications [43]. Briefly, 50 ng of WT SARS-CoV-2 S1 protein or mutated SARS-CoV-2 S1 protein was coated in 100 μL per wells on ELISA plates (2592, Costar, USA) overnight at 4 °C followed by washing 3 times with phosphate-buffered saline with 0.05% Tween 20 (PBST). The ELISA plates were incubated for 1 h at 37 °C in 100 μL per well blocking buffer (PBST containing 5% FBS). Then, for 1 h at 37 °C, 100 μL of fivefold serially diluted parental mAbs or Bi-Nab or unrelated control anti-human PD-1 IgG in blocking buffer was added to each well. After 3 washes with PBST, the plate was incubated with HRP-conjugated anti-human IgG antibody for 1 h at 37 °C followed by washing with PBST. An addition of 50 μL of 3,30,5,50-tetramethyl-benzidine (TMB) (T0440, Sigma‒Aldrich, USA) buffer was added per well and reacted at 25 °C for 5 min and then stopped by 0.2 M H_2_SO_4_ stop buffer. The optical density (OD) value was measured at 450 nm using an iMark microplate absorbance reader (BIO-RAD, USA), and nonlinear regression was used to calculate the concentration for 50% of the maximal effect (EC_50_).

### ELISA-based RBD-ACE2-binding inhibition assay

As previously described [2], 200 ng of human ACE2 protein per well was coated on ELISA plates at 4 °C overnight followed by washing 3 times with PBST. The ELISA plates were blocked with 100 μL of blocking buffer per well at 37 °C for 1 h as above, meanwhile, fivefold serial dilutions of parental mAbs or a cocktail or Bi-Nab were incubated with 4 ng/mL SARS-CoV-2 RBD-mouse Fc protein for 1 h at 25 °C. Then, the incubated mixture was added to the blocked ELISA plates and incubated at 37 °C for another 1 h. The anti-mouse Fc HRP antibody was used to detect the binding SARS-CoV-2 RBD with 1 h incubation at 37 ℃. After washing, TMB substrate was added to the ELISA plates, and the reaction was stopped with a 0.2 M H_2_SO_4_ stop buffer. Absorbance at 450 nm was recorded by a microplate reader.

### Antibody binding to cell surface-expressed coronavirus spike proteins

HEK293T cells were transfected with 2 μg of plasmids separately encoding different coronavirus S proteins using linear polyethylenimine (PEI) (408,727, Sigma‒Aldrich) at 80–90% confluence in 6-well cell culture plates. DNA-PEI complexes were made at a ratio of 1:2 in 1 mL DMEM, and the transfection mixture was added to cell culture plates after 15 min of incubation at room temperature. After transfection for 4–6 h at 37 °C, the medium was changed to complete cell growth medium. Cells were detached by 1 mM EDTA after 40 h of transfection and washed with cold PBS containing 2% FBS to remove EDTA. The cells were incubated with 5 μg/mL mAb 35b5 or mAb 32C7 or Bi-Nab or ACE2 for 1 h on ice followed by FITC-conjugated anti-human IgG or FITC-conjugated anti-His IgG for 1 h on ice away from light. Finally, the cells were analyzed by flow cytometry (BD Biosciences, USA) and FlowJo 10.

### SARS-CoV-2 pseudovirus neutralization assay

The pseudovirus neutralization assay was previously described [3]. In brief, HEK293T cells were transfected with pLenti-GFP, psPAX2 and SARS-CoV-2 S plasmids at a ratio of 1:1:1 by using PEI. After transfection for 4–6 h, the cells were refreshed with a complete cell growth medium and incubated for another 40 h. At the same time, HEK293 cells were transfected with plasmid encoding hACE2 for 36 h followed by seeding into 24-well plates the day before transduction with pseudovirus. HEK293T culture supernatants containing SARS-CoV-2 pseudovirus were harvested and mixed with fivefold serially diluted mAb or a cocktail or Bi-Nab for 1 h, and the 500 μL per well mixture was incubated with human ACE2-expressing HEK293 (hACE2/293) cells overnight and changed to fresh complete cell growth medium with another 40 h of incubation. The luciferase activity of SARS-CoV-2-type pseudovirus-infected hACE2/293 cells was detected by the Dual-Luciferase Reporter Assay System (E2520, Promega, USA).

### Statistical analysis

The statistical analyses were performed using GraphPad Prism 9.0 software. Unless otherwise stated, the data represent the results of three or two independent experiments. The mean values ± SEMs and the EC_50_ values in the ELISA binding assay were calculated by using sigmoidal dose‒response nonlinear regression. The mean values ± SEMs and the IC_50_ values in the ELISA-based RBD-ACE2 binding inhibition assay and each pseudovirus neutralization assay were determined by the nonlinear four-parameter dose‒response curve.

## Data Availability

Data are contained within the article.
